# Discrepancy between invasive and non-invasive blood pressure readings in extremely preterm infants in the first four weeks of life

**DOI:** 10.1371/journal.pone.0209831

**Published:** 2018-12-28

**Authors:** Tobias Werther, Lukas Aichhorn, Sigrid Baumgartner, Angelika Berger, Katrin Klebermass-Schrehof, Ulrike Salzer-Muhar

**Affiliations:** 1 Division of Neonatology, Pediatric Intensive Care and Neuropediatrics, Department of Pediatrics and Adolescent Medicine, Medical University of Vienna, Vienna, Austria; 2 Division of Pediatric Cardiology, Department of Pediatrics and Adolescent Medicine, Medical University of Vienna, Vienna, Austria; Umea University Hospital, SWEDEN

## Abstract

**Background:**

The agreement between invasive and non-invasive blood pressure (BP) readings in the first days of life of preterm infants is contentiously debated.

**Objective:**

To compare mean, systolic and diastolic invasive (IBP) and non-invasive BP (NBP) readings obtained during routine care in the first four weeks of life of extremely preterm infants.

**Methods:**

We extracted pairs of IBP and NBP readings obtained from preterm infants born below 28 weeks of gestation from the local database. After exclusion of erroneous measurements, we investigated the repeated measures correlation and analyzed the agreement (bias) and precision adjusted for multiple measurements per individual.

**Results:**

Among 335 pairs of IBP and NBP readings obtained from 128 patients, we found correlation coefficients >0.65 for mean, systolic and diastolic BP values. The bias for mean BP readings was -0.4 mmHg (SD 6.1), for systolic BP readings 6.2 mmHg (SD 8.1), and for diastolic BP readings -4.3 mmHg (SD 6.5). Overestimation of systolic IBP and underestimation of diastolic IBP by the non-invasive measurement were found both in the group with gestational age from 23 to 25.9 weeks and in the group with gestational age from 26 to 27.9 weeks. Systolic NBP readings tended to exceed invasive readings in the range <50 mmHg (bias 9.9 mmHg) whereas diastolic NBP readings were lower than invasive values particularly in the range >30 mmHg (bias -5.5 mmHg).

**Conclusion:**

The disagreement between invasive and non-invasive BP readings in infants extends to the first four weeks of life. Biases differ for mean, systolic and diastolic BP values. Our observation implies that they may depend on the range of the blood pressure. Awareness of these biases and preemptive concomitant use of IBP and NPB readings may contribute to reducing over- or under-treatment.

## Introduction

In extremely preterm infants, continuous blood pressure (BP) monitoring via an arterial line immediately after birth remains standard [1]. Arterial lines are also placed when preterm infants are critically ill not only for BP monitoring but also for repeated blood withdrawal. In this fragile population, the insertion of an arterial catheter is not always feasible or sometimes an indwelling peripheral catheter has to be removed because of low perfusion of the distal tissue [2, 3]. Then BP is determined by the non-invasive oscillometric technique and neonatologists at the bedside will be concerned by a bias between invasive (IBP) and non-invasive (NBP) readings. The few studies on the agreement between invasive blood pressure (IBP) and non-invasive blood pressure (NBP) readings in the early life of preterm infants report partly inconsistent results [4–8]. They only considered mean BP [4–6, 8] and/or were restricted to the first days of life [4, 6–8].

Blood pressure measurement guides therapeutic intervention in the neonatal intensive care unit (NICU). The mean BP thresholds used to trigger an intervention affect the achieved BP and inotrope usage [9]. Systolic BP is used to estimate pulmonary pressure in the echocardiographic assessment of early pulmonary hypertension in extremely preterm infants [10]. Diastolic BP is considered to reflect the intravascular blood volume and a drop in the diastolic BP is an alarming sign for loss of volume [11].

The purpose of this comparison study was to analyze correlation, agreement and precision relating to IBP and NBP readings obtained during routine care in the first four weeks of life in preterm infants born below 28 weeks of gestation.

## Materials and methods

The local ethics committee (Ethikkommission der Medizinischen Universität Wien) approved the study (EK Nr: 2044/2016). The need for individual consent was waived (data were analyzed anonymously).

### Study population

In a retrospective observational study, we included all preterm infants admitted at our NICU between October 2011 and December 2015 born below 28 weeks of gestation. Infants with congenital heart disease were excluded.

### Invasive blood pressure measurement

Preterm infants born below 28 weeks of gestational age routinely received a peripheral arterial line shortly after birth and in situations of critical illness when continuous BP monitoring and regular blood samples were required. For the peripheral arterial line (PAL), either a 24G or a 26G catheter (Neoflon Cannula, length 19 mm, BD Infusion Therapy AB, Helsingborg, Sweden) was used. Only physicians were allowed to insert arterial lines, using an aseptic technique. After insertion, the arterial line was maintained by a continuous infusion of a heparinized isotonic saline solution (0.3–1 mL/h). Eliminating air bubbles and blood clots from the catheter-transducer system made sure that the pressure wave was not damped. For calibration, the invasive transducer (TruWave pressure transducer, Edwards Lifesciences, CA) was zeroed at the level of the right atrium. An indwelling arterial line was removed whenever continuous BP monitoring had no further benefit compared to non-invasive measurements and regular blood sampling was no longer required, or if it was not functioning and/or hypo-perfusion of distal tissue was observed. IBP readings were automatically recorded every fifteen minutes in the local information system database ICCA (IntelliSpace Critical Care and Anesthesia, Phillips, NL).

### Non-invasive blood pressure measurement

Neonatal cuffs (NBP Cuff Neo Size 1–3, Dräger Medical GmbH, Lübeck, Germany) were used for the non-invasive oscillometric BP measurements. The cuff size was chosen according to the manufacturer’s recommendations and was levelled to the infant’s right atrium. The NBP measurements were obtained in the upper arm or in the lower leg, based upon ease of access. BP measurements were performed with the Infinity Delta XL Patient Monitor System (Dräger, Lübeck, Germany) which transferred the data to the local database.

### Study protocol

In our NICU, we have no standard procedure that specifies when to take a NBP reading during invasive BP measurements. Whenever an arterial line was in place, the decision to obtain a NBP reading was left to the primary care team. Most often, NBP readings were used to report reliability of both IBP and NBP readings. Using an electronic query bound to the patient cohort, we extracted invasive and non-invasive BP readings, corresponding time of measurement, site of measurement, the insertion and removal time of the arterial line as well as patient baseline characteristics and administered medications from the information system database. Data were imported in the computing environment Matlab R2015b (The MathWorks, Natick, MA, USA) where we performed subsequent queries, data visualization and statistical computations. We identified all episodes of IBP readings for each patient and searched for NBP readings in the corresponding timeframe. For each NBP sample, we chose the IBP reading that preceded the NBP reading with the shortest time gap. This was done as the non-invasive measurement might alter subsequent IBP readings [12]. We allowed a maximal time gap of fifteen minutes between NPB and corresponding IBP.

**Fig 1:**
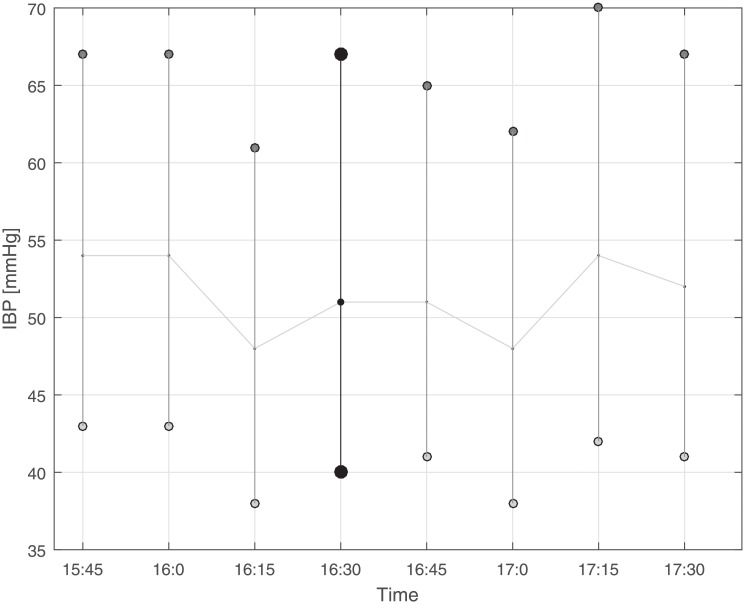
Illustration of invasive blood pressure (IBP) readings (approximately one hour before and one hour after the non-invasive blood pressure (NBP) reading considered for analysis) as it was used to screen IBP tracks for potential artifacts or inconsistency. Since the changes in this example were only moderate (less than 10 mmHg), the IBP marked with black circles was selected for analysis. Note that the NBP reading was not visualized in the figure in order to blind selection/exclusion of IBP readings.

**Fig 2:**
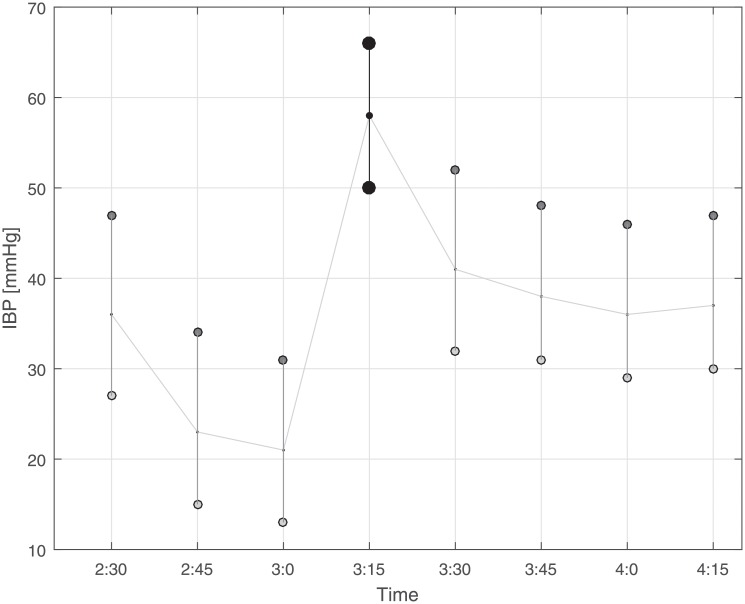
Illustration of invasive blood pressure (IBP) readings (approximately one hour before and one hour after the non-invasive blood pressure (NBP) reading considered for analysis) as it was used to screen IBP tracks for potential artifacts or inconsistency. Since the changes in this example were more than 10 mmHg in particular after the IBP under consideration (marked with black circles), the pair corresponding to this IBP reading was excluded from analysis. Note that the NBP reading was not visualized in the figure in order to blind selection/exclusion of IBP readings.

The following safety precautions were defined to exclude redundant and erroneous measurements. 1. Incorrect NBP readings: We observed that systolic NBP values of 102 or 107 mmHg (usually they occurred in a clustered form) resulted from incorrect readings, probably due to an inappropriate application of the cuff. Pairs with such systolic values were excluded. 2. Readings with BP amplitudes (difference between systolic and diastolic value) smaller than five mmHg: these readings were excluded as they very likely indicated damped recordings. 3. IBP readings exhibiting sudden changes and episodes with fluctuations: We screened one-hour episodes prior to and after the NBP reading of each BP pair by visual inspection, and excluded all those pairs exhibiting changes in the baseline of the IBP. In detail, we excluded episodes with one of the following criteria: at least two changes of approximately more than 10 mmHg of the mean IBP between two adjacent recordings (restless state) or a constant change of approximately more than 10 mmHg of the mean IBP after the IBP under consideration (recalibration of arterial line suspected). It is important to mention that the NBP values of the BP pair did not appear in the graphical presentation of the IBP tracing. For the sceening procedure, we build a simple graphical user interface (in MATLAB) that visualized only the IBP tracing and allowed to deselect episodes with fluctuations in the IBP recordings as described. Examples are presented in Figs 1 and 2. 4. Multiple IBP readings paired with a single IBP reading: We permitted only one NBP reading for each IBP considered for analysis and excluded all multiple NBP readings that were recorded within 15 minutes of the IBP reading under consideration except for the closest in time.

### Data analysis

Correlations between IBP and NBP readings were analyzed using the repeated measures correlation (rmcorr implemented in R version 3.3.3) that accounts for non-independence among multiple observations per individual. We calculated agreement (bias, mean difference) and precision (1.96 SD of the difference corresponding to the 95% limits of agreement in the Bland-Altman plots) adjusting for multiple observations per individual [13]. We applied the Bland-Altman plots to depict the patterns of discord between IBP and NBP. Data were evaluated for different periods (28 days, week 1 versus week 2–4), two different groups of gestation (group I, from 23+0 to 25+6/7 weeks of gestation, versus group II, from 26+0 to 27+6/7 weeks of gestation) and for local BP intervals. We compared parametric data using the Student t-test, non-parametric data using the Mann-Whitney-U test and binary data using the chi-square test. A p-value below 0.05 was considered significant.

## Results

A total of 350 preterm infants born at 23+0 to 27+6/7 weeks of gestation and admitted to our NICU from October 2011 to December 2015 were enrolled in this study. Four infants with congenital heart disease were excluded. Among the remaining 346 infants, 335 (97%) had an indwelling PAL for various periods within the first four weeks of life.

**Fig 3:**
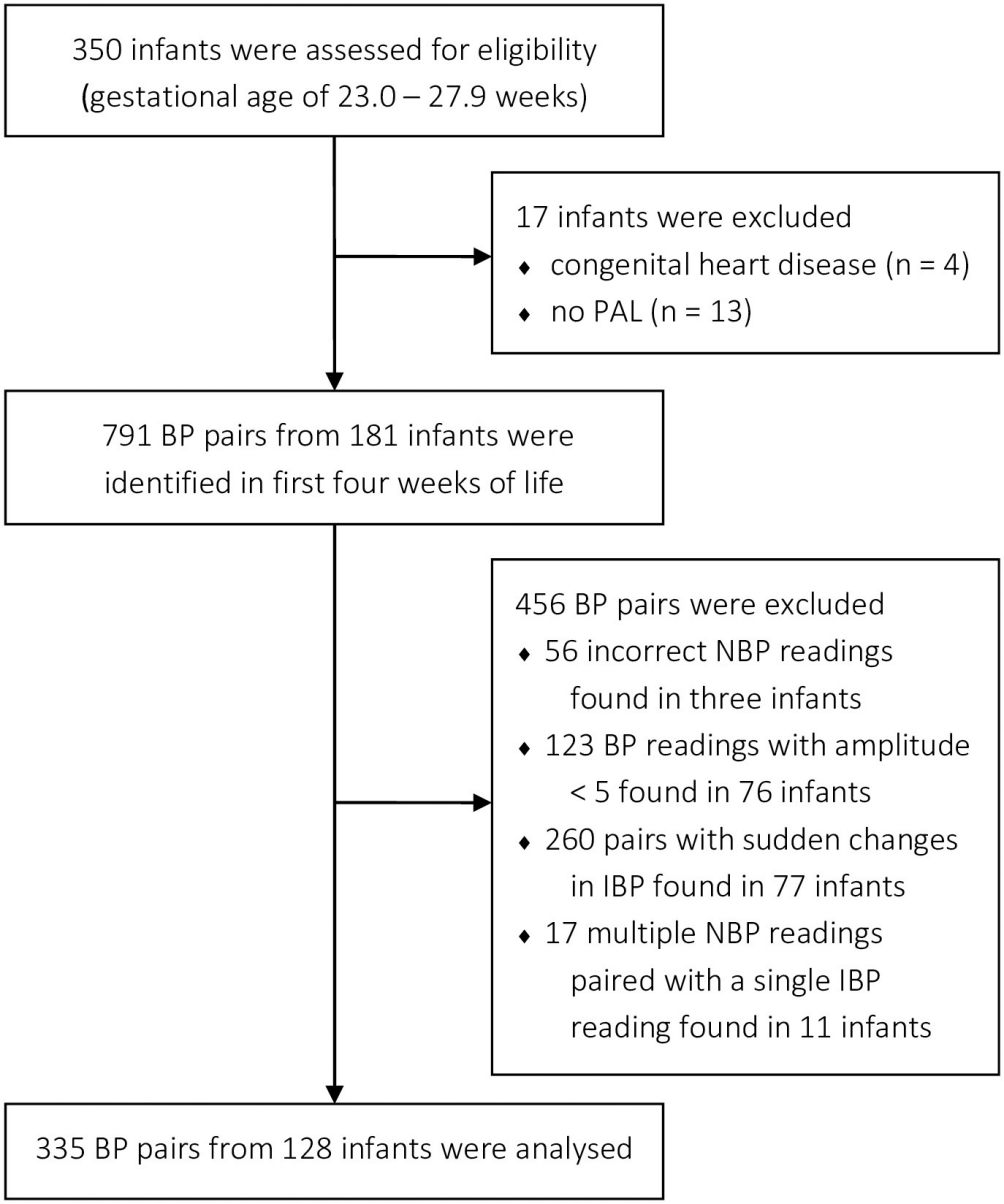
Eligibility and exclusion of infants and pairs of invasively and non-invasively blood pressure readings (BP, blood pressure; NBP, non-invasive blood pressure; IBP, invasive blood pressure; PAL, peripheral arterial line).

In total, we could identify 791 pairs of IBP and NBP readings obtained from 181 patients. After excluding pairs with suspected incorrect NBP readings (n = 56), pairs with small BP amplitudes (n = 123), pairs exhibiting changes in the baseline of the IBP (n = 260), and multiple NBP readings matched with the same IBP reading (n = 17), the number of pairs considered for analysis decreased to 335. These pairs were obtained from 128 different patients (Fig 3). In this population, gestational age (median 25.6 weeks) and birth weight (mean 751 g) did not differ significantly from the overall cohort. However, administration of inotropic and sedating agents was more frequent in the examined population. Table 1 reports the characteristics of the overall cohort and the examined population. No arrhythmia was documented. In the examined cohort, 76 infants contributed only one BP pair, and ten infants contributed more than five BP pairs that were considered for analysis. The mean time difference between IBP and NBP reading for the 335 BP pairs amounted to 7.1 min (SD 4.1).

**Table 1:** Baseline characteristics.

Patients’ characteristics	Cohort		p-Value ^a^
	overall	examined	
Number, [n]	346	128	-
Gestational age in weeks, median (range)	25.8 (23.0 27.9)	25.6 (23.1 27.9)	0.23
Weight in g, mean (SD)	781 (200)	752 (192)	0.15
Males	59	62	0.59
Survival beyond 28 days of life	86	88	0.70
At least one PAL during first 4 weeks of life	96	100	0.03
Two consecutive PALs during first 4 weeks of life	20	29	0.04
More than one PALs during first 4 weeks of life	40	63	< 0.01
Inotropes^1^ during first four weeks of life	49	66	< 0.01
Morphine^2^ during first four weeks of life	52	69	< 0.01
**PALs in first 4 weeks of life**			
Total, [n]	575	277	-
Left radial artery	41	36	0.13
Right radial artery	33	35	0.47
Left ulnar artery	4	3	0.19
Right ulnar artery	3	2	0.30
Left brachial artery	2	2	0.78
Right brachial artery	2	2	0.67
Left tibial posterior artery	6	6	0.66
Right tibial posterior artery	9	14	0.02

PAL, Peripheral arterial line; SD, standard deviation

Data are presented as percentage unless otherwise indicated.

^a^ Student t-test for birth weight, Mann-Whitney-U for gestational age, chi-square test for all other variables.

### NBP versus IBP

**Fig 4:**
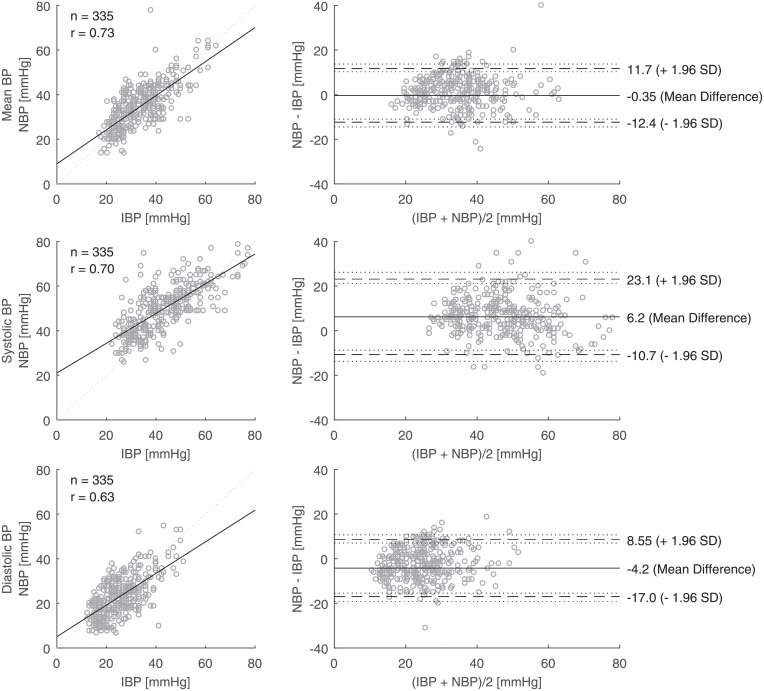
Linear regressions (left; dotted line: Line of equality; r: Pearson correlation coefficient) and Bland-Altman plots (right; dashed lines: Limits of agreement; dotted lines: Confidence intervals) for pairs (n = 335) of invasively (IBP) and non-invasively measured blood pressure (NBP) readings in the first four weeks of life obtained from preterm infants born below 28 weeks of gestation.

**Fig 5:**
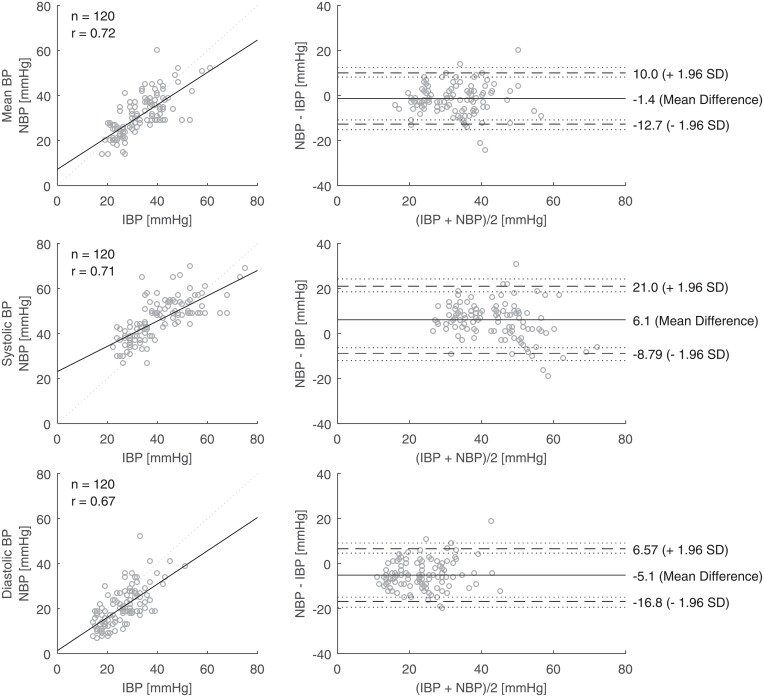
Linear regressions (left; dotted line: Line of equality; r: Pearson correlation coefficient) and Bland-Altman plots (right; dashed lines: Limits of agreement; dotted lines: Confidence intervals) for pairs (n = 120) of invasively (IBP) and non-invasively measured blood pressure (NBP) readings in the first weeks of life obtained from preterm infants born below 28 weeks of gestation.

Values of NBP are plotted against IBP for the entire time of examination in Fig 4. For the mean BP, the linear line of best fit was close to the line of equality. The bias was -0.4 mmHg and the precision 12.0 mmHg (n = 335, Table 2). For the systolic BP, the non-invasive method gave higher readings than the invasive measurement by 6.2 mmHg on average. For the diastolic BP, the non-invasive method gave lower readings than the invasive measurement by 4.3 mmHg on average. Similar patterns were found for each gestational group (Table 2). We also plotted the values of NBP against IBP from the first week of life, as this time of examination was used in most previous studies (Fig 5).

**Table 2:** Results.

GA	Period	Patients/ Pairs, n	BP	Mean IBP [mmHg]	Bias [mmHg]	Precision (95%CI) [mmHg]	Rm (95%CI)
**23+0–27+6**							
	28 days	128/335	mean	34.61	-0.35	12.0 (10.6 14.1)	0.78 (0.72 0.83)
			systolic	43.51	6.21	16.9 (15.0 18.2)	0.69 (0.61 0.75)
			diastolic	27.44	-4.21	12.8 (11.2 16.2)	0.67 (0.59 0.74)
	week 1	73/120	mean	32.68	-1.36	11.4 (9.5 13.9)	0.62 (0.40 0.85)
			systolic	40.23	6.11	14.9 (12.4 18.2)	0.53 (0.29 0.71)
			diastolic	26.11	-5.14	11.7 (9.8 14.3)	0.53 (0.29 0.71)
	week 2–4	71/215	mean	36.31	0.50	11.8 (10.0 14.5)	0.80 (0.73 0.85)
			systolic	46.44	6.19	17.3 (14.8 21.3)	0.68 (0.58 0.76)
			diastolic	28.63	-3.53	13.3 (11.2 16.2)	0.69 (0.60 0.77)
**23+0–25+6**							
	28 days	73/189	mean	33.04	0.45	11.2 (9.5 13.6)	0.84 (0.77 0.90)
			systolic	41.64	6.81	17.0 (14.4 20.8)	0.76 (0.67 0.83)
			diastolic	26.35	-3.43	10.7 (9.1 13.0)	0.75 (0.66 0.82)
	week 1	31/50	mean	28.50	-0.19	9.8 (7.4 13.3)	0.76 (0.46 0.91)
			systolic	35.36	7.31	14.9 (11.2 20.3)	0.65 (0.26 0.85)
			diastolic	22.93	-3.96	10.4 (7.7 14.1)	0.77 (0.47 0.91)
	week 2–4	50/139	mean	35.32	0.68	11.7 (9.6 14.7)	0.84 (0.77 0.90)
			systolic	44.95	6.44	17.2 (14.1 21.8)	0.75 (0.65 0.83)
			diastolic	28.00	-3.24	11.2 (9.2 14.1)	0.75 (0.64 0.83)
**26+0–27+6**							
	28 days	55/146	mean	36.71	-1.40	12.7 (10.5 15.9)	0.70 (0.57 0.79)
			systolic	45.99	5.41	16.7 (13.9 20.9)	0.58 (0.42 0.70)
			diastolic	28.90	-5.24	14.8 (12.1 18.5)	0.59 (0.43 0.71)
	week 1	42/70	mean	35.77	-2.22	12.1 (9.6 15.7)	0.55 (0.22 0.77)
			systolic	43.83	5.23	14.8 (11.6 19.2)	0.45 (0.09 0.71)
			diastolic	28.46	-6.00	12.3 (9.8 16.0)	0.45 (0.08 0.71)
	week 2–4	21/76	mean	38.65	0.06	12.2 (9.0 17.4)	0.74 (0.58 0.84)
			systolic	50.00	5.60	17.5 (13.1 24.7)	0.58 (0.37 0.73)
			diastolic	30.13	-4.23	17.3 (12.3 25.2)	0.61 (0.41 0.75)

BP, Blood pressure; CI Confidence interval; IBP, Invasively measured blood pressure; GA, Gestational age; Rm, Repeated measures correlation coefficient.

### Bias based on IBP range

The bias of the non-invasive readings seemed to vary, depending on the range of the IBP (Table 3). For the mean BP, the bias was highest in the upper range (>40 mmHg) with absolute values close to three mmHg. For the systolic BP, the bias was highest in the lower range (<35 mmHg) with values close to 10 mmHg and lowest in the upper range (>50 mmHg) with values close to one mmHg. For the diastolic BP, the bias was highest in the upper range (>30 mmHg) with values close to -6 mmHg. The results were similar for both groups of gestation (S1 Table).

**Table 3:** Results.

**Mean blood pressure**			
**Range [mmHg]**	**Patients/ Pairs [n]**	**Bias [mmHg]**	**Precision (95%CI) [mmHg]**
entire	128/335	-0.35	12.0 (10.6, 14.1)
< 30	67/146	1.01	10.7 (8.8, 13.1)
30–40	58/117	0.92	12.6 (10.4, 15.7)
> 40	50/72	-2.52	12.0 (9.5, 15.3)
**Systolic blood pressure**			
**Range [mmHg]**	**Patients/ Pairs [n]**	**Bias [mmHg]**	**Precision (95%CI) [mmHg]**
entire	128/335	6.21	15.9 (15.0, 20.0)
< 35	58/95	9.89	15.6 (12.7, 19.5)
35–50	70/151	7.24	15.5 (13.0, 19.0)
> 50	52/89	1.37	14.6 (11.9, 18.4)
**Diastolic blood pressure**			
**Range [mmHg]**	**Patients/ Pairs [n]**	**Bias [mmHg]**	**Precision (95%CI) [mmHg]**
entire	128/335	-4.21	12.8 (11.2, 14.9)
< 20	48/99	-2.31	11.2 (8.9, 14.3)
20–30	66/146	-3.52	11.3 (9.4, 13.8)
> 30	64/90	-5.45	14.9 (12.2, 18.4)

Difference of invasive and non-invasive blood pressure readings in preterm infants (gestational age < 28 weeks) for different blood pressure ranges. (CI, Confidence interval).

## Discussion

We performed a comparison study to determine the bias of non-invasive readings of mean, systolic and diastolic BP in the first four weeks of life of 182 extremely preterm infants. The three main findings of this study can be summarized as follows:

First, the bias of the mean BP values was small as reported in other studies [4, 7, 8], which is reassuring for clinical practice. In our study, the bias remained small too for low mean IBP values (<30 mmHg). This is in contrast to the findings by Takci et al. who reported that the bias of the mean BP increased to 6.5 mmHg for IBP readings below 30mmHg [8]. Takci et al. obtained their data from multiple measurements in a relatively small group of study participants (n = 27) and did not specify a correction for multiple measurements per individual which may add a systematic error to the comparison.

Second, our results show that the non-invasive method leads to over-reading of the systolic IBP while it leads to under-reading of the diastolic IBP. This is in line with Lalan et al. who reported a mean difference of 8.3 mmHg and -4.3 mmHg for systolic and diastolic BP readings in newborns with PALs, respectively [14].

Third, the results indicated that the non-invasive systolic BP was approximately 10 mmHg higher when the invasive measurement was lower than 35 mmHg and only 1.5 mmHg higher when the invasive measurement was higher than 50 mmHg. For the diastolic BP readings, the bias was slightly higher (approximately -5.5 mmHg) in the upper range (diastolic IBP >30 mmHg). These findings, albeit being remarkable, have only observational character and need to be confirmed in further studies. At the bedside, this over-reading of systolic BP by non-invasive measurements could lead to under- or over-treatment, for instance, in the management of early pulmonary hypertension in preterm infants [15].

We found relatively high values of precision (1.96 SD range 9.8–17.5 mmHg, Table 2), reflecting the individual variability between IBP and NBP readings. Such high variability was also described by Koenig et al., who reported a bias of the mean BP from -1.2 mmHg (SD 6.1) to 3.5 mmHg (SD 6.7) in infants with less than 1000 g and an umbilical arterial line (UAL) [5]. In a similar cohort, Meyer et al. found a better precision (bias -0.36, 2 SD 6.5 mmHg) of the non-invasive mean BP in the first 24 hours of life [4]. Takci et al. found a small difference between the mean IBP and NBP readings (bias 0.02 mmHg) in the first week of life in 27 newborns including 21 very low birth weight infants and reported a precision as high as 16.7 mmHg (1.96 SD) [8]. Similar precision values were also found in critically ill children [16], which indicates, that the variability of the BP measurements is independent of the size of the vessels.

The reasons for the reported variations in BP measurements in preterm infants are manifold. As to inaccurate NBP measurement, the cuff size has a large impact and a small cuff tends to overestimate BP [17]. In our NICU, the nurse staff is trained to use the respective appropriate cuff. However, in clinical practice, the limb circumference often is only estimated. Also using both upper and lower limbs for NBP measurements might increase the range of variation, as limits of agreement up to 20 mmHg have been reported when comparing the location of non-invasive measurements [6]. Koenig et al. found a bias of 3.5 mmHg comparing the right arm mean BP versus UAL mean BP, and a bias of -1.2 mmHg for the right leg versus UAL measurements in preterm infants with birth weight smaller than 1000g [5]. They suggested that the lower limb should be preferred for NBP readings in preterm infants. The location of the arterial line may also account for variations in the IBP readings. Recent studies provided contradictory results when comparing IBP readings derived from either PALs or UALs. Meyer et al reported that the degree of agreement was not affected by the position (UAL versus PAL) of the catheter [4], thereby confirming former results [18], whereas Lalan et al. found a greater bias in the mean BP for invasive measurements from the radial artery (4.8 mmHg) than for measurements from UALs (0.4 mmHg) [14]. Sources of inaccuracy with invasive BP measurements are air bubbles and blood clots in the arterial line causing damping with low systolic and high diastolic readings. In addition, the small diameter of the catheter acts as a low-pass filter, resulting in under-reading of systolic blood pressure [19, 20].

In clinical practice it is not only important to know all those potential sources of variation and different readings with non-invasive and invasive BP measurement, but also to keep in mind, that the intra-arterial BP measurement, which is considered the “gold standard”, and the oscillometric measurement are based on entirely different principles. In the former, the pressure waveform of the arterial pulse is transmitted via a column of fluid to a pressure transducer where it is converted into an electrical signal, which is processed, amplified and converted into a visual display by a microprocessor. In the latter, the cuff is automatically inflated to a preset value. Reducing the inflation gradually, the pressure wave of the arterial pulse causes oscillations in the vessel, which can be detected by the cuff. Mean arterial pressure corresponds to the maximum of oscillations and an algorithm applied to the change of oscillations sets systolic and diastolic arterial pressure values [21]. These different approaches are the rationale behind the results of this study as variations between these two methods primarily originate from the principle of operation rather than from inaccuracy.

Finally, similar findings have been obtained in other intensive care scenarios with children and adults [16, 22–24]. The decision, which BP monitoring is used, needs to be tailored to the individual patient’s risk in the clinical setting [16, 25]. The same holds true for the preterm infant in the NICU. In critical situations, when positive inotropic or vasodilator agents are administrated, the use of noninvasive BP measurements should supplement the invasive readings to target specific BP goals [24] and might give a better understanding of the discrepancy between the two methods in the individual patient which is particularly helpful when the invasive measurement needs to be abandoned.

### Strengths and limitations

Our study has several limitations. The retrospective study design is prone to bias and confounding errors. However, the relatively large number of individuals might reduce sources of bias and confounding. The individual decisions of the nurses and clinicians in charge to take a NBP reading during continuous IBP measurement might entail a notable risk of bias, in particular, when recalibration of the arterial line after discrepant NBP readings resulted in a change in IBP measurements. We tried to reduce this risk of bias by excluding all pairs with changes of approximately more than 10 mmHg in the mean IBP after the NBP reading. An important source of error results from motion artifacts. As we had no information about the individual infant’s resting phase, we used the fluctuations in the invasive BP readings as a respective indicator and excluded pairs with highly fluctuating invasive readings. The time-difference within each BP pair between IBP and NBP might add another bias. This study was unable to meet the rigorous criteria of a research laboratory setting, but the findings highlight the real-world clinical assessment of BP in a high volume NICU. We analyzed neither ventilator nor inotropic support. Lalan et al. did not find any effect of the ventilator or inotropic support on the agreement between IBP and NBP readings [14]. We did not differentiate between pre-ductal and post-ductal measurements, as we could not find any substantial difference in invasive BP readings obtained from pre- and post-ductal PALs when correcting for gestational age and day of life. The strengths of the study were the careful visual screening for artifacts and manipulation of the invasive BP readings, the study period of four weeks and the inclusion of critically ill preterm infants.

## Conclusion

Non-invasive and invasive BP readings disagree in the first four weeks of life of extremely preterm infants. The bias is least for the mean BP. Our observation that the bias may be range-dependent for the systolic and diastolic BP needs further confirmation. Non-invasive systolic BP is over-read and non-invasive diastolic BP is under-read, which is explained by the underlying principle of the oscillometric method. Our findings can support neonatologists in their correct evaluation of non-invasive BP readings, should they have to abandon arterial lines. The preemptive use of non-invasive BP measurements to supplement invasive BP readings may reduce subsequent inappropriate interventions by improving understanding of the non-invasive BP readings.

## Supporting information

S1 Data Data. Anonymized data of invasive and non-invasive blood pressure pairs (n = 791) that were automatically extracted from the local database with additional information on site of peripheral arterial line, time gap between invasive and non-invasive measurements, time of measurement, gestational age, and inclusion for analysis.
(XLSX)

S1 Table Results for two groups of gestational age. Difference of invasive and non-invasive blood pressure readings for different blood pressure ranges and groups of gestational age in the first 28 days of life of extremely preterm infants (CI, Confidence interval; GA, Gestational age).
(DOCX)
