# Supplementary material for: Discrepancy between invasive and non-invasive blood pressure readings in extremely preterm infants in the first four weeks of life
Source: PLoS One. 2018 Dec 28;13(12):e0209831. doi: 10.1371/journal.pone.0209831 (PMC6310249; doi:10.1371/journal.pone.0209831)
Supplement: S1 Table — Difference of invasive and non-invasive blood pressure readings for different blood pressure ranges and groups of gestational age in the first 28 days of life of extremely preterm infants (CI, Confidence interval; GA, Gestational age). (DOCX) [file pone.0209831.s002.docx]

**S1 Table. Results for two groups of gestational age.**

| **Mean blood pressure** | | | | | |
| --- | --- | --- | --- | --- | --- |
|  | **GA** | **Range [mmHg]** | **Patients/ Pairs [n]** | **Bias [mmHg]** | **Precision (95%CI) [mmHg]** |
|  | **23+0 - 25+6** | entire | 73/189 | 0.45 | 11.2 (9.5 13.6) |
|  |  | < 30 | 47/96 | 1.75 | 9.7 (7.8 12.4) |
|  |  | 30 - 40 | 31/61 | 0.93 | 12.1 (9.6 16.4) |
|  |  | > 40 | 23/32 | -1.96 | 11.0 (7.8 16.0) |
|  | **26+0 - 27+6** | entire | 55/146 | -1.40 | 12.7 (10.5 15.9) |
|  |  | < 30 | 20/50 | -0.37 | 12.2 (8.4 18.1) |
|  |  | 30 - 40 | 27/56 | 0.92 | 13.6 (9.9 19.0) |
|  |  | > 40 | 27/40 | 3.00 | 12.9 (9.4 18.0) |
| **Systolic blood pressure** | | | | | |
|  | **GA** | **Range [mmHg]** | **Patients/ Pairs [n]** | **Bias [mmHg]** | **Precision (95%CI) [mmHg]** |
|  | **23+0 - 25+6** | entire | 73/189 | 6.81 | 17.0 (11.4 20.8) |
|  |  | < 35 | 40/65 | 9.51 | 15.1 (11.8 19.7) |
|  |  | 35 - 50 | 36/79 | 7.53 | 16.0 (12.5 21.1) |
|  |  | > 50 | 26/45 | 1.48 | 15.2 (11.5 21.3) |
|  | **26+0 - 27+6** | entire | 55/146 | 5.41 | 16.7 (13.9 20.9) |
|  |  | < 35 | 18/30 | 10.71 | 17.0 (11.5 25.8) |
|  |  | 35 - 50 | 34/72 | 6.93 | 15.0 (11.6 20.0) |
|  |  | > 50 | 26/44 | 1.27 | 14.3 (10.4 20.1) |
| **Diastolic blood pressure** | | | | | |
|  | **GA** | **Range [mmHg]** | **Patients/ Pairs [n]** | **Bias [mmHg]** | **Precision (95%CI) [mmHg]** |
|  | **23+0 - 25+6** | entire | 73/189 | -3.43 | 10.7 (9.1 13.0) |
|  |  | < 20 | 32/72 | -1.76 | 10.8 (8.2 14.5) |
|  |  | 20 - 30 | 39/73 | -3.05 | 9.9 (7.9 13.0) |
|  |  | > 30 | 33/44 | -4.71 | 13.4 (10.1 18.1) |
|  | **26+0 - 27+6** | entire | 55/146 | -5.24 | 14.8 (12.1 18.5) |
|  |  | < 20 | 16/27 | -3.40 | 12.2 (8.0 19.3) |
|  |  | 20 - 30 | 27/73 | -4.21 | 13.1 (9.7 18.1) |
|  |  | > 30 | 31/46 | -6.24 | 16.3 (12.4 22.3) |

Difference of invasive and non-invasive blood pressure readings for different blood pressure ranges and groups of gestational age in the first 28 days of life of extremely preterm infants (CI, Confidence interval; GA, Gestational age).
